# Insect abundance patterns on vertebrate remains reveal carrion resource quality variation

**DOI:** 10.1007/s00442-022-05145-4

**Published:** 2022-03-16

**Authors:** Blake M. Dawson, James F. Wallman, Maldwyn J. Evans, Philip S. Barton

**Affiliations:** 1 Centre for Sustainable Ecosystem Solutions, School of Earth, Atmospheric and Life Sciences, University of Wollongong, Wollongong, NSW Australia; 2 Faculty of Science, University of Technology Sydney, Ultimo, NSW Australia; 3 Fenner School of Environment and Society, Australian National University, Canberra, ACT Australia; 4 Department of Ecosystem Studies, Graduate School of Agricultural and Life Sciences, The University of Tokyo, Tokyo, Japan; 5 Future Regions Research Centre, Federation University Australia, Mount Helen, VIC, Australia

**Keywords:** Carcass, Decomposition, Necrobiome, Succession

## Abstract

Resource quality is a key driver of species abundance and community structure. Carrion is unique among resources due to its high nutritional quality, rapidly changing nature, and the diverse community of organisms it supports. Yet the role resource quality plays in driving variation in abundance patterns of carrion-associated species remains poorly studied. Here we investigate how species abundances change with a measure of resource change, and interpret these findings to determine how species differ in their association with carrion that changes in quality over time. We conducted field succession experiments using pigs and humans over two winters and one summer. We quantified the effect of total body score, an objective measure of resource change, on adult insect abundance using generalised additive models. For each species, phases of increasing abundance likely indicated attraction to a high-quality resource, and length of abundance maxima indicated optimal oviposition and feeding time. Some species such as the beetle *Necrobia rufipes* had a rapid spike in abundance, suggesting a narrow window of opportunity for carrion resource exploitation, while species like the wasp *Nasonia vitripennis* had a gradual change in abundance, indicating a wide window of resource exploitation. Different abundance patterns were also observed between species occurring on pigs and humans, suggesting cadaver type is an important aspect of resource quality. Our findings show that species abundances, unlike species occurrences, can reveal additional detail about species exploitation of carrion and provide information about how resource quality may drive competition and variation in insect community succession.

**Supplementary Information:**

The online version contains supplementary material available at 10.1007/s00442-022-05145-4.

## Introduction

Resource quality is a key driver of species abundance and diversity, often having a direct influence on competition and predation within ecological communities (Lucas et al*.* 2009; Marcarelli et al. 2011; Ochoa-Hueso et al. 2019). For consumption-related resources such as food, resource quality is usually defined by the nutritional output provided by the resource (Hladyz et al. 2009). Resources of higher nutritional value often attract a higher abundance and diversity of species; a result driven by individuals seeking greater metabolic return on foraging investment, leading to enhanced survival and reproduction outcomes (Ober and Hayes 2008; Price et al. 2016; Vaudo et al. 2018). The influence of resource quality and its effects on species abundances have been studied extensively in numerous ecological communities such as pollinator and leaf litter communities (Rantalainen et al. 2004; Sileshi and Mafongoya 2007; Fowler et al. 2016). But there has been little study of resource quality and its effect on carrion communities, despite the implicit acknowledgement of the rapid change in carrion quality underpinning the very well-documented patterns in succession (Michaud et al. 2015; Barton and Evans 2017; Evans et al. 2020; Dawson et al. 2021b).

Carrion is unique among resources due to its very high nutritional quality and its ephemeral nature (Barton et al. 2013, 2019). Carrion is able to support a wide range of species including vertebrate apex and meso-scavengers, and an enormous diversity of invertebrates and microorganisms, all coexisting on a shared resource and collectively forming the ‘necrobiome’ (Benbow et al. 2019). Both interspecific and intraspecific competition for resources on carrion is intense due to the high number of species exploiting the remains (Charabidze et al. 2021). One of the key mechanisms allowing coexistence of carrion species is temporal partitioning, where species will utilise carrion at different times over the course of decomposition, which leads to successive waves of carrion use and colonisation (Hanski 1987; Ives 1991). Carrion-dependent species are generally highly specialised and adapted to locating decomposing remains and determining when carrion resource quality is optimal for feeding or ovipositing behaviour (Evans et al. 2020).

The quality of carrion as a resource is difficult to define due to its dynamic and continuously changing state, which includes changes in quality (nutritional output), digestibility, and quantity (biomass) (Barton et al. 2019; Benbow et al. 2019). The objective quality of carrion varies greatly from the onset of fresh decay through to dried remains, with optimal nutritional quality differing among species and depending on their adaptations and timing of colonising the remains (Dawson et al. 2021b). For some arthropods, such as primary colonising flies that require carrion in this condition for their larvae to feed on, fresh carrion is preferred as a high-quality resource (Evans et al. 2020). By contrast, carrion at this initial stage is likely undesirable and of poor quality for necrophagous beetles that specialise on dried remains for feeding (Battán Horenstein and Linhares 2011; Caballero and León-Cortés 2014). The resource quality of carrion is therefore a species-specific concept and likely a key driver of the dynamics of carrion insect community succession, diversity and abundance.

Recent research on carrion insects has demonstrated that changes in carrion resource, as indicated by total body score (TBS), is an important factor driving insect species richness and community turnover (Dawson et al. 2021b). Total body score is an ideal measure of resource change as it is a measure of decomposition and incorporates both the physical and chemical changes occurring on carrion. Importantly, using TBS allows researchers to incorporate the continuous and dynamic change occurring in carrion, thereby enabling more complex carrion insect community succession models to be developed (Schoenly and Reid 1987; Michaud et al. 2015). However, to determine how resource quality is influencing carrion insects, individual species abundance patterns need to be examined. This is because resource quality is a species-centric and species-specific concept, and assessment of abundance patterns against an external and objective measure of resource change provides a way to assess the quality of the resource as preferred by different species.

Examining the abundances of species at carrion may reveal additional details about their ecology and interactions than would be shown examining their occurrences alone. Abundances are more likely to reveal the species’ optimal time of resource utilisation during carrion decomposition, and can provide insights into coexistence mechanisms occurring on carrion by showing non-overlapping abundances (Barton and Evans 2017). For example, we suggest a phase of increasing abundance might indicate preferred high resource quality, whereas a phase of decreasing abundance might indicate undesirable or waning resource quality and reduced oviposition or feeding potential. This type of information is potentially lost when analysing only species richness or occurrence data. Studies of insect species’ abundances tend to focus on the forensic applications through minimum post-mortem interval (mPMI) estimations by examining species patterns in relation to time (Matuszewski et al. 2010), or they assess abundance changes in relation to thermal summation models such as accumulated degree days (ADD) (Michaud and Moreau 2009). The few studies that have examined species abundances within an ecological context have shown abundances varying among seasons and habitat types (Benbow et al. 2013; Barton et al. 2017; Engasser et al. 2021). Using an objective measure of resource change, such as TBS, allows comparisons of abundance patterns to be made among habitats, seasons, and potentially carrion types.

The aim of this study was to identify how different species of the carrion insect community respond to change in carrion as it decomposes as a shift in resource quality over time. To do this, we examined the relationship between adult insect species abundance patterns and an objective measure of carrion resource change using the total body score (TBS) metric. Total body score is a semi-quantitative measure of resource change that incorporates both the physical and chemical changes occurring on carrion, and therefore the change in nutritional value of the remains (Megyesi et al*.* 2005). We focused on examining abundance patterns of the most common species of three dominant insect taxa found at carrion: Diptera, Coleoptera and Hymenoptera. We tested the following hypotheses:

1. Abundance of Diptera (flies) species will be high at low TBS values. This is because fresh carrion would likely be of higher quality to primary colonising species therefore driving high abundance numbers (Frederickx et al. 2012).
2. Coleoptera (beetles) species will exhibit high abundance at high TBS values. This is either because some beetles prey upon other carrion insects, and therefore will not arrive until prey has colonised carrion, while other beetles feed on dried remains and will not arrive until late in the decomposition process (Watson and Carlton 2005).
3. Hymenoptera (ants and wasps) abundance will have no association with TBS. This is because ants are generalist and opportunistic species that are present throughout decomposition (Eubanks et al. 2019), while wasps are parasitic and dependent on host abundance (Voss et al. 2009).

## Materials and methods

### Study site

We conducted three field insect community succession experiments using human cadavers and pig carcasses over two winters (‘Winter A’: 30th May–15th October 2018 and ‘Winter B’: 8th May–2nd October 2019) and one summer (‘Summer’: 9th November–16th December 2019). We aimed to replicate seasons to account for yearly variation but unfortunately a second summer experiment could not be conducted in 2018 due to human cadaver unavailability. Experiments were completed once no noticeable decomposition changes were occurring and insect activity was minimal, resulting in the disparity in length between the winter and summer experiments as decomposition was slower during winter. All experiments took place at the Australian Facility for Taphonomic Experimental Research (AFTER), operated by the University of Technology Sydney (UTS). The site is 4.86 hectares and surrounded by dry sclerophyll *Eucalyptus* forest with scattered rural housing in the nearby vicinity. We recorded on-site measurements of ambient temperature and humidity every fifteen minutes using a HOBO MX2302 Ext temperature and relative humidity data logger protected by a solar radiation shield.

### Human and pig set-up and experimental design

We used six human cadavers and five pig carcasses (see Dawson et al. 2021b for human and pig details). Our sample size is consistent with other studies using human cadavers, since sourcing cadavers in high numbers is logistically challenging (Knobel et al. 2019). The cadavers were donated to AFTER through the UTS Body Donation Program, approved by the UTS Human Research Ethics Committee Program Approval (UTS HREC REF NO. ETH15-0029). Domestic pigs (*Sus scrofa*) were purchased post-mortem from a licensed abattoir, therefore requiring no ethics approval in accordance with the Australian Code of Practice for the Care and Use of Animals for Scientific Purposes (2004). Pigs were killed by the abattoir using a captive head bolt and were transported to AFTER within one hour of death. All human cadavers were kept refrigerated after death and delivered to AFTER within 48 h.

Once at AFTER, 5 m × 5 m plots were selected within the facility and human cadavers were placed on their backs within the plots. Due to licencing agreements, pigs were placed along the outside of the facility on their sides. To maximise entomological independence between replicates, we placed pigs a minimum of 20 m apart from one another, and at least 100 m away from any human cadavers. Entomological independence within the facility among human cadavers was difficult to achieve; therefore, cadavers were placed closest to other cadavers that had already skeletonised or become desiccated, with little or no observable entomological activity. All pigs and humans were placed on a metal mesh on top of the soil, apart from pigs and humans in Winter A which were placed directly on the soil. Pigs and humans placed on metal mesh were infrequently lifted off the ground for a few seconds throughout the decomposition process to collect weight-loss data for another study. We selected semi-shaded areas for pig and human placement and protected them from vertebrate scavenging animals by enclosing the pigs and humans in scavenger proof metal cages. We placed four pitfall traps filled with a mix of propylene glycol and 70% ethanol evenly around each pig and human following the layout described by Dawson et al. (2020).

On each sampling day we collected both adult and larval carrion insects using a combination of standardised methods including timed sweep netting and manual sampling, as well as pitfall traps. We sampled between 11:00 and 13:00 h and documented decomposition progress and photographed the remains using a Canon EOS 70D camera with a 18–55 mm STM lens. We sampled once every day for the first week, once every second day for the following three weeks, and then gradually reduced our sampling frequency depending on the progress of decomposition and level of insect activity. All adult insects and larvae were identified to species level where possible using morphological keys and molecular techniques, as described by Dawson et al. (2020).

### Data analysis

To determine how abundance patterns differed with resource change for common carrion insects, we used total body score (TBS) as a proxy for resource change, using the method by Megyesi et al*.* (2005). TBS is a commonly used and objective measure of decomposition state and indicator of resource change (Nawrocka et al. 2016; Connor et al. 2017; Roberts et al. 2017; Dawson et al. 2021b). We applied a separate numeric value based on the physical decomposition of the remains to each body region (head, torso and limbs) and combined these values together to provide an overall score of the decomposition rate. Across the 11 cadavers (six humans and five pigs), we recorded 336 TBS points. We used those Diptera, Coleoptera and Hymenoptera species that had a total abundance of > 200 across all experiments as these species were deemed the most common carrion insects and modelling of species requires a minimum abundance for statistically rigorous models to be constructed. Exceptions to this were four *Calliphora* species (*Calliphora augur*, *Calliphora hilli hilli*, *Calliphora ochracea* and *Calliphora stygia*), which we grouped together into one ‘*Calliphora’* species group for modelling. We grouped these species due to their importance as early colonisers. We also grouped Phorids together into one ‘Phoridae’ species group as identifying them to genus and species level was difficult due to limited taxonomic resources available for this family. In total, we used 18 species and species groups for analysis, comprising 22,780 insects, including nine groups of Diptera (11,974), five of Coleoptera (5445) and four of Hymenoptera (5361).

To analyse abundance patterns, we fitted generalised additive models (GAMs), using TBS as our predictor variable and species abundance as our response variable. We used GAMs because the species abundance relationships over the decomposition process of carrion were most likely to be non-linear (Moreau 2021). We pooled all abundance data from each experiment together and fitted GAMs for each individual species or species group. In each GAM, we used species abundance as the response variable and TBS as the smoothed predictor variable with cadaver type (pig versus human) as a multiplier in the smoothed term. We assumed a Poisson error distribution with a logarithmic link function for each model, unless a fit was over dispersed, in which case we assumed a negative binomial error distribution. Only TBS values in the range 3–24 were used for modelling as not all pigs and humans reached a TBS above 24. By truncating our data to within this range we were able to provide a full set of comparisons among all cadavers in winter and summer. We measured humidity and rainfall, but these factors were not included in our analysis as they did not vary between experiments (Supp. Table S1). We also recorded ambient temperature and accumulated degree days (ADD), which varied at each TBS value though this variation was consistent between pigs and humans (Supp. Fig. S1). There was also seasonal variation in ambient temperature (See Dawson et al. 2021a). Temperature influences decomposition progress, but TBS is a standardised decomposition metric, which can account, in part, for seasonal and temperature differences (Dawson et al. 2021b). We conducted all modelling and graphing using the R software program (R Core Team 2019) using the mgcv (Wood 2017) and ggplot2 (Wickham 2016) packages.

## Results

### Diptera

**Fig. 1:**
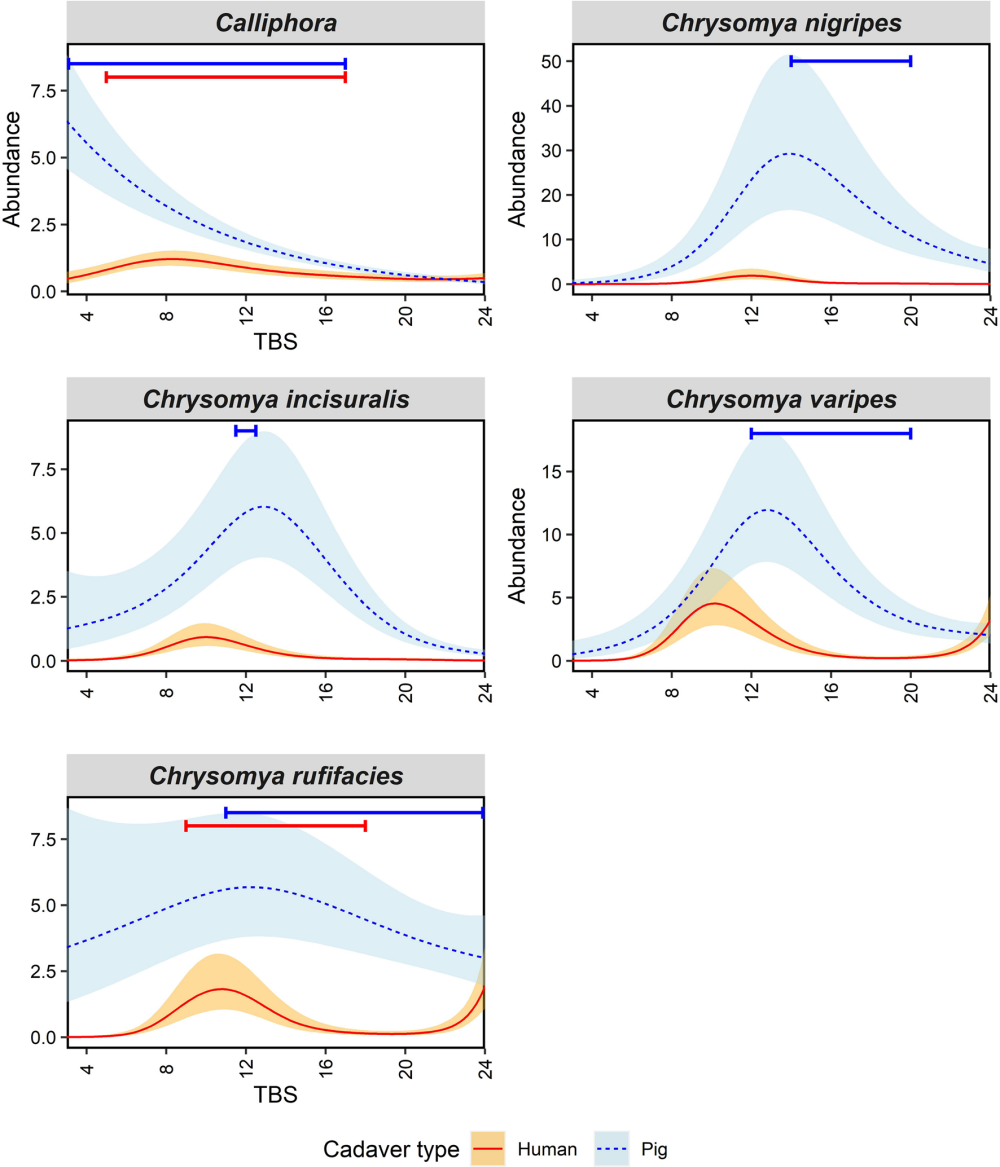
Generalised additive model plots modelling mean adult calliphorid abundance over total body score (TBS). Blue dotted lines represent mean adult abundance for pigs and red solid lines for humans. Shaded error bands represent standard errors of adult means. Coloured bars along the top of the plots display the minimum and maximum TBS when larvae of that species were collected on pigs (blue, above) and humans (red, below)

For species in the family Calliphoridae, GAM results showed that most species were significantly associated with TBS on humans, except for the *Calliphora* species group (Supp. Table S2). Similarly, most species’ abundances were significantly associated with TBS on pigs, except for *Chrysomya rufifacies*. The percent deviance explained by the models varied between 18.6 and 45.2%, with *Chrysomya nigripes* having the highest. The abundance patterns of *Ch. nigripes* and *Chrysomya incisuralis* were similar, with both displaying a large spike in adult abundance during mid decomposition on pigs only, with few adults present on humans (Fig. 1). Larvae of *Ch. nigripes* were present on pigs after the initial spike, while larvae of *Ch. incisuralis* were present slightly before the spike (Fig. 1).

Adults of *Chrysomya varipes* and *Ch. rufifacies* also shared similar patterns, with a small spike in adult abundance occurring early in decomposition for humans, and a larger spike occurring on pigs slightly later in decomposition (Fig. 1). The abundance spike was more prolonged for *Ch. rufifacies* than *Ch. varipes*. Adult abundance on humans for both species increased again towards the higher TBS values in late decomposition. Larvae on pigs and humans were first recorded for both species during the first abundance spike, except for *Ch. varipes* where no larvae were recorded on humans. The *Calliphora* species group, unlike the other calliphorids in this study, showed no mid decomposition spike in adult abundance (Fig. 1). Instead, the abundance of this group on pigs peaked during initial decomposition and slowly decreased, while on humans it displayed low adult abundance throughout decomposition. Despite this group’s low adult abundance, larvae were present from the beginning of decomposition until mid to late decomposition.

**Fig. 2:**
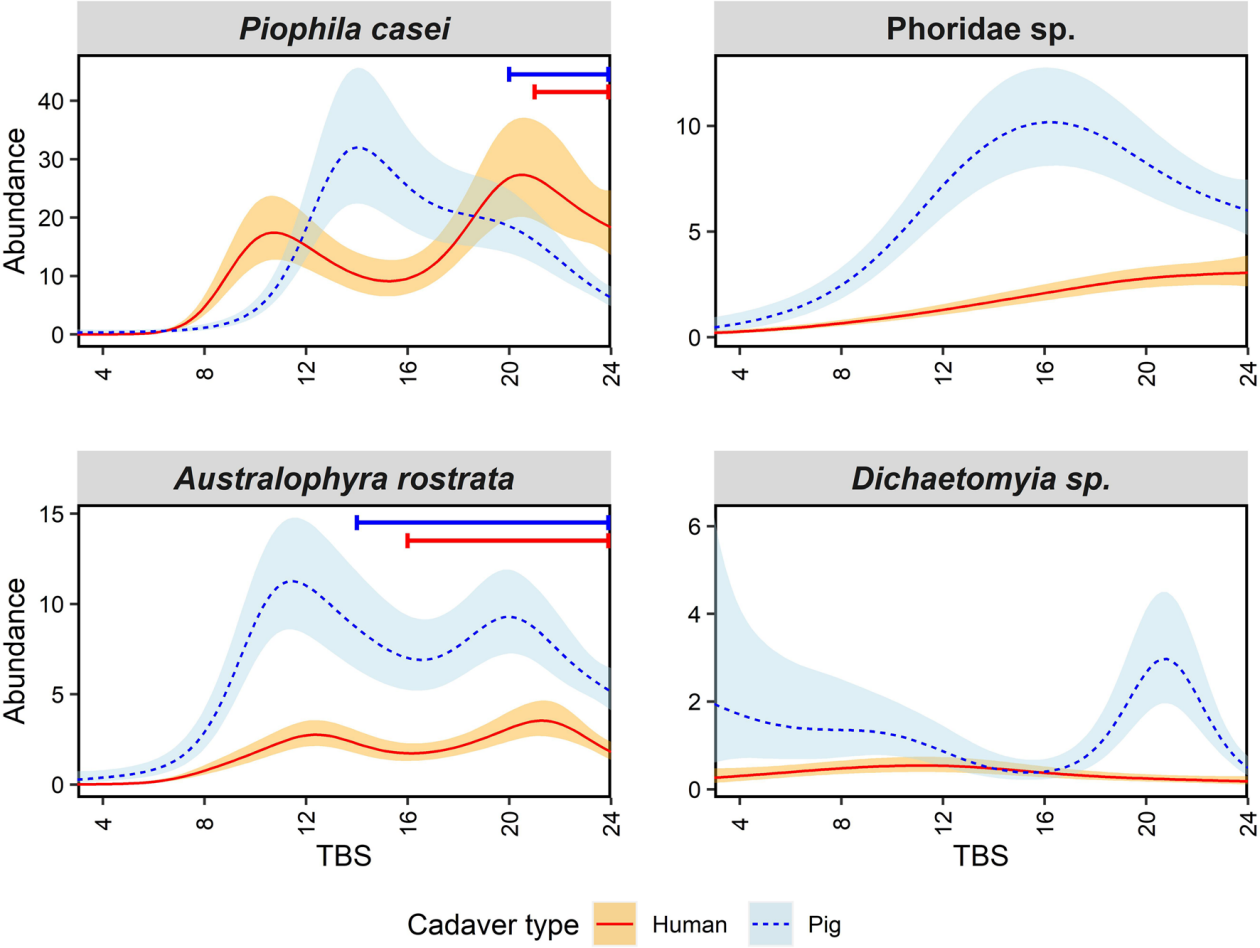
Generalised additive model plots modelling mean adult Diptera (excluding calliphorids) abundance over total body score (TBS). Blue dotted lines represent mean adult abundance for pigs and red solid lines for humans. Shaded error bands represent standard errors of adult means. Coloured bars along the top of the plots display the minimum and maximum TBS when larvae of that species were collected on pigs (blue, above) and humans (red, below)

For all other Diptera, the models revealed that all species’ abundances on pigs were significantly associated with TBS, while almost all species’ abundances on humans were significantly associated with TBS, with the exception of *Dichaetomyia* sp. (Muscidae) (Supp. Table S2). The percent deviance explained by the models varied between 20.1 and 35.7%, with *Australophyra rostrata* (Muscidae) having the highest. *Piophila casei* (Piophilidae) showed a difference in adult abundance patterns between pigs and humans as the pigs had one abundance peak and the humans had two, with neither peak occurring at the same TBS values (Fig. 2). Despite adult differences, the presence of larvae was similar between pigs and humans, with larvae first recorded well after adult abundances first peaked. The Phoridae species group also displayed different patterns of adult abundance between pigs and humans, with the group on pigs exhibiting one drawn out spike, while on humans the group displayed a linear increase in abundance as TBS increased (Fig. 2).

*Australophyra rostrata* also had two distinct spikes of abundance on pigs but on humans had a more flattened abundance relationship with TBS (Fig. 2). The larval activity of *A. rostrata* was similar between carcass types despite the adult abundance differences, with larvae not observed until after the first abundance spike. For *Dichaetomyia* sp., pigs displayed varied abundance throughout decomposition with a spike in abundance at higher TBS values, while the species was absent from humans (Fig. 2).

### Coleoptera

**Fig. 3:**
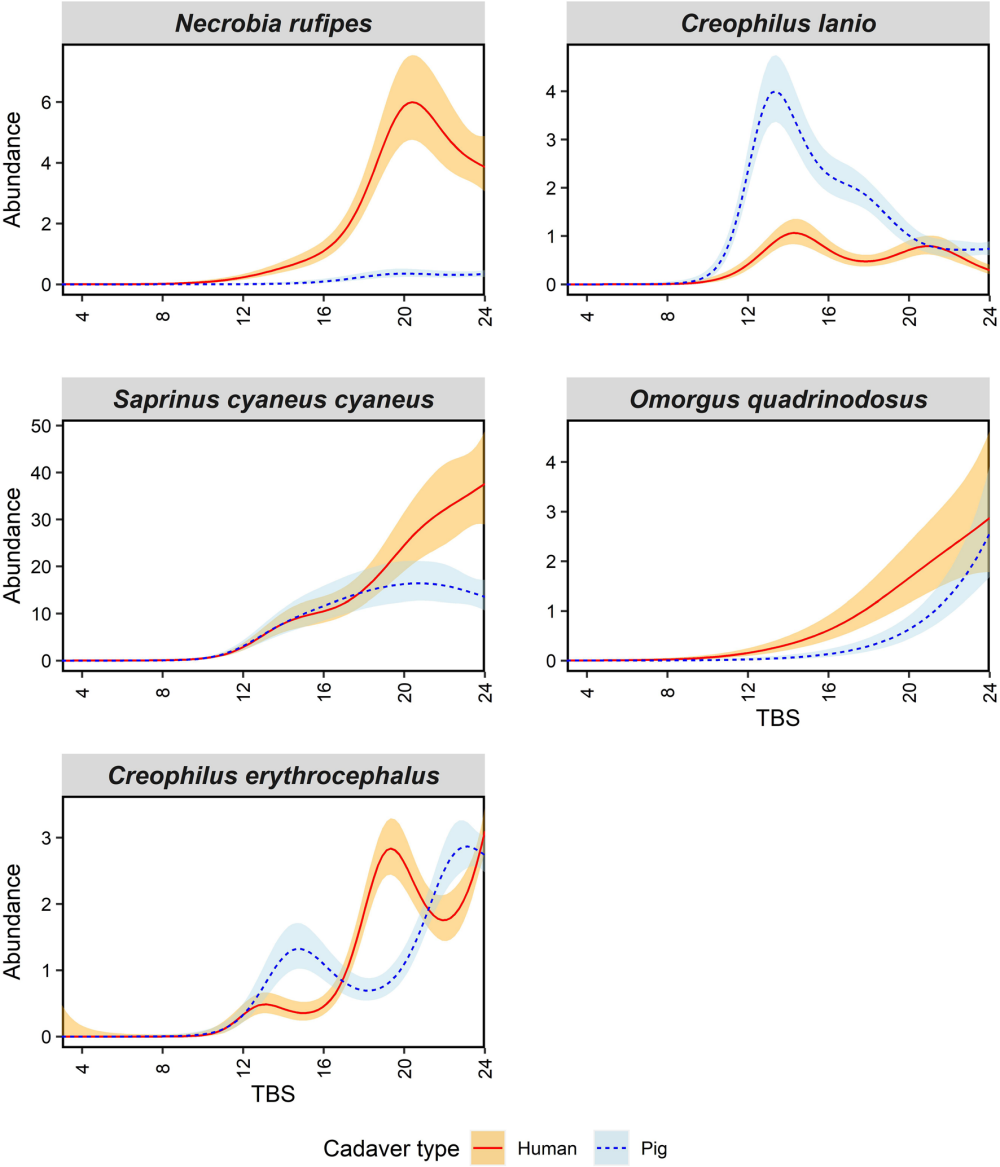
Generalised additive model plots modelling mean adult Coleoptera abundance over total body score (TBS). Blue dotted lines represent mean adult abundance for pigs and red solid lines for humans. Shaded error bands represent standard errors of adult means. No Coleoptera larvae were collected

For species within the order Coleoptera, we found almost all species’ abundances to be significantly associated with TBS on both pigs and humans, with the exception of *Necrobia rufipes* (Cleridae) on pigs (Supp. Table S2). The percent deviance explained by the models varied between 37 and 64.3% with *N. rufipes* having the highest. The models for all species are similar in that there were no early spikes in adult abundance, with adults not arriving until around 11 TBS on both pigs and humans. *Necrobia rufipes* was found to have a large spike in adult abundance on pigs towards the end of decomposition, while they were mostly absent on pigs (Fig. 3). Conversely, *Creophilus lanio* (Staphylinidae) displayed a large adult abundance spike on pigs, but limited abundance on humans (Fig. 3). We found *Saprinus cyaneus cyaneus* (Histeridae) and *Omorgus quadrinrodosus* (Trogidae) to share similar adult abundance patterns between pigs and humans, as both gradually increased as TBS increased, with initial adult arrival occurring at the same time (Fig. 3). By contrast, for *S. cyaneus cyaneus*, abundance continued to rise on humans but gradually decreased on pigs. *Creophilus erythrocephalus* displayed the most volatile adult abundance patterns with multiple spikes occurring on both pigs and humans, but at different TBS values (Fig. 3).

### Hymenoptera

**Fig. 4:**
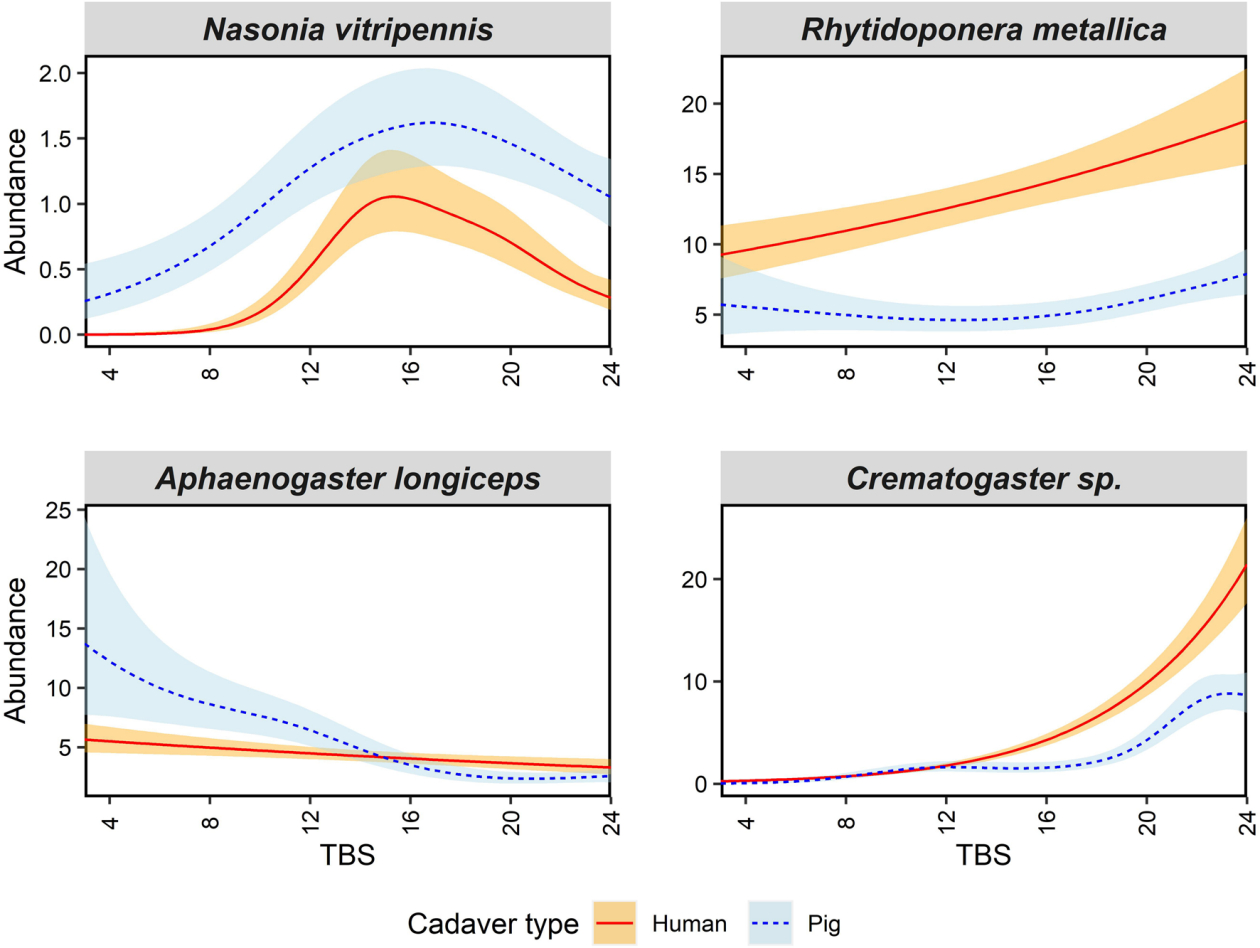
Generalised additive model plots modelling mean adult Hymenoptera abundance over total body score (TBS). Blue dotted lines represent mean adult abundance for pigs and red solid lines for humans. Shaded error bands represent standard errors of adult means. No Hymenoptera larvae were collected

For Hymenoptera, we found that some species abundances were significantly associated with TBS, except for *Rhytidoponera metallica* (Formicidae) and *Nasonia vitripennis* (Pteromelidae) on pigs and *R. metallica* and *Aphaenogaster longiceps* (Formicidae) on humans. The percent deviance explained by the models varied between 6.6 and 38.2% with *Crematogaster* sp. (Formicidae) having the highest (Supp. Table S2). The hymenopteran models are mostly different from those for Diptera and Coleoptera models in that they lack spikes in adult abundance; rather, they either gradually increase or decrease with TBS in a linear nature. The exception to this is *N. vitripennis* which displayed a gradual spike during mid decomposition at the same time on both pigs and humans (Fig. 4). *Aphaenogaster longiceps* adult abundance started high then decreased quickly on pigs, while on humans the adult abundance stayed relatively stable (Fig. 4). *Rhytidoponera metallica* displayed the opposite pattern with a steady abundance increase on humans but relatively stable abundance on pigs. Lastly, we found *Crematogaster* sp. adult abundance patterns to be very similar between pigs and humans with a rapid increase in abundance as TBS increased.

### Relative abundance patterns

**Fig. 5:**
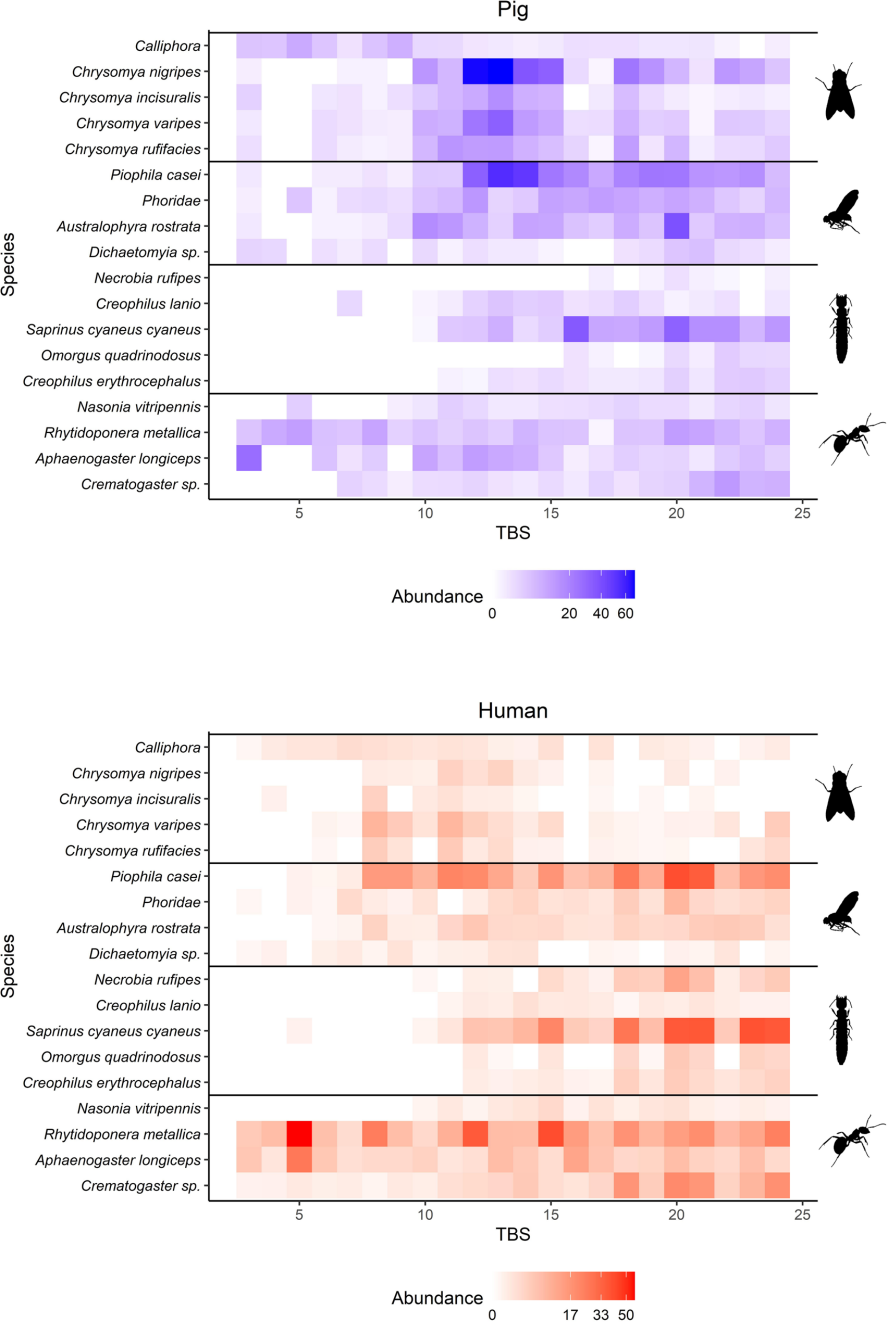
Heat map depicting changes in mean adult abundance with increasing TBS for each species and species group. Blue represents pigs and red for humans

Clear successional patterns emerged when viewing the abundance of individual species and species groups across the whole community (Fig. 5). These patterns were most obvious for Diptera and Coleoptera, with *Calliphora* species arriving first, followed by other Diptera species, and Coleoptera species arriving last. Most Diptera arrived in large numbers in association with mid TBS scores, although some arrived earlier in lower abundance, particularly on pigs. The Coleoptera displayed greatest abundances at higher TBS scores with almost none present during low TBS scores. Hymenoptera species displayed no clear successional pattern as they arrived first in high numbers on both pigs and humans and remain in relatively stable populations throughout decomposition. In general, most species were more abundant and arrived earlier on pigs than humans, as seen with the *Chrysomya* and the other mid–late stage Diptera species.

## Discussion

We aimed to determine how species of carrion insects perceive resource change as a shift in resource quality over time by examining adult abundance patterns on vertebrate remains. We found that the abundance of insects was significantly associated with resource change for almost all insect species. Our results provide insight into how each species responds to carrion resource change, as some species displayed gradual changes in abundance, indicating large windows of preferred high resource quality. By contrast, other species displayed rapid peaks in abundance indicating short windows of preferred high resource quality. Below, we discuss how each taxonomic group responds to resource change and how cadaver type relates to resource quality.

### Adult abundances reveal timing of high resource quality

As hypothesised, *Coleoptera* species did not arrive on either pigs or humans until mid TBS values and either had a rapid peak in abundance upon initial arrival, or a gradual increase in abundance. A rapid increase and decrease was observed for the omnivorous clerid *N. rufipes* and the predatory staphylinid *C. lanio*, which suggests that these species have a narrow window of optimal carrion exploitation and preferred resource quality. This narrow window of optimal resource quality suggests that these species might be under strong intraspecific competitive pressure as they are constrained to a narrow niche (Kocárek 2001). This contrasts with other Coleoptera species such as the predatory histerid *S. cyaneus cyaneus* and necrophagous trogid *O. quadrinodosus*, which displayed a sustained, gradual increase in abundance after initial arrival on carrion. This behaviour indicates that these species have a larger window of optimal resource quality for colonising carrion, where carrion quality is high for a longer period. This gradual colonisation behaviour suggests competition for resources is less intense compared with those species that display rapid peaks in abundance (Braack 1987). This is because the resource used by these species, whether it be the carrion or prey species, is optimal during decomposition for a longer period, therefore likely leading to less competition. For example, *Dermestes maculantus* has a narrow feeding preference, consuming skin and fleshy tissue, while other species such as *Trox* spp. avoid interspecific competition by having a wider feeding preference (Braack 1987). Our results are similar to other studies that examined Coleoptera arrival patterns in relation to coarser decay stages, finding that most species delay arriving until active decay (Sharanowski et al. 2008; Battán Horenstein and Linhares 2011; Castro et al*.* 2013; Weithmann et al. 2021). The narrow window of peak abundance of the clerid *N. rufipes*, and its strong association with resource change, suggest that this species could provide more accurate PMI estimations than species with peak abundance spread over a wider window.

The Diptera displayed a mixed association with resource change, with most species arriving as predicted, such as *Calliphora* species arriving first, followed by *Chrysomya* species. The larvae of *Chrysomya* species were first recorded during the phase of decrease after adult abundances peaked. Oviposition, therefore, likely occurred around peak adult abundance and not when adults first arrived at carrion. Adults therefore arrived at carrion when resource quality is less then optimal and wait until resource quality is high before ovipositing. Being secondary colonisers, *Chrysomya* larvae likely prefer carrion to be in a particular state of decomposition to allow for optimal growth and development. This is facilitated by modification of the carrion resource by primary colonising flies such as *Calliphora* species. In this case, the resource quality required by the larvae might reflect both the nutritional output as well as the digestibility of the carrion. However, previous research has suggested that the process of facilitation does not adequately apply to carrion succession (Michaud and Moreau 2017). Alternatively, delayed oviposition may be the result of priority effects, as some *Chrysomya* larvae such as *Ch. rufifacies* are facultative predators of other Diptera larvae (Brundage et al. 2014). Therefore, *Chrysomya* larvae may gain additional fitness benefits if colonising carrion after other Diptera species as the *Chrysomya* larvae gain an additional resource for consumption. Delayed oviposition may also relate to one or more other factors such as lack of optimal oviposition sites (Archer and Elgar 2003), requirement of a protein meal before ovary development in females (Avancini and Linhares 1988), density requirements of larvae for optimal maggot mass growth (Charabidze et al. 2011; Johnson and Wallman 2014), or selective mating behaviour (Beehler and Foster 1988; Jones et al. 2014).

The other Diptera families generally displayed complex series of both increasing and decreasing phases of abundance during carrion decomposition, with some species displaying multiple peaks in abundance. This complex abundance pattern was most evident for the muscid *A. rostrata* and piophilid *P. casei*, with larvae first observed towards the end of decomposition, well after initial adult arrival. Carrion resource quality for these species, unlike others, may have multiple points where quality is high, rather than a single point as observed in the calliphorids and Coleoptera. Despite being known late-stage colonisers, *A. rostrata* and *P. casei* arrived relatively early at low TBS values, indicating that they are likely able to locate carrion using chemical cues similar to those employed by primary and secondary colonising flies (Frederickx et al. 2012). Previous studies have reported that these species generally do not arrive until competition for resources is less intense, or the remains are in an optimal condition for larvae to feed upon. For some piophilids, the latter may occur when fatty acids are present (Martín-Vega 2011). However, like in our study, other studies have observed these species arriving early in decomposition (Barton et al. 2017) and arriving in successive waves, with oviposition not recorded until later waves (Voss et al. 2011). These results highlight the complex species–resource interactions occurring on carrion and the role resource quality plays in carrion insect community succession.

Hymenoptera species either gradually decreased or increased in abundance in a relatively linear relationship as decomposition progressed, with only the parasitic pteromalid *N. vitripennis* displaying a gradual spike during decomposition. This gradual spike indicates a large window of optimal resource quality for *N. vitripennis*, which, rather than feeding directly on the remains, parasitises Diptera larvae (Steiner et al. 2006). The other Hymenoptera in our study are generalist Formicidae (ant) species that were present in the local environment and likely using carrion to either prey on other insects around the remains, exploit the moisture present in decomposition fluids, or feed on the epidermal skin layer of the carrion (Barton et al. 2017; Eubanks et al. 2019; Evans et al. 2020). Resource quality is likely not a specific factor in driving the adult abundance of Hymenoptera. This abundance is probably more influenced by hymenopteran populations and the proximity of nests in the local environment.

### Cadaver type influence on species abundance

Adult abundance for most insect species generally varied in two distinct ways, which suggests species are responding differently to the resource quality of the two cadaver types. First, species were observed arriving at the same time, but at different magnitudes, with pigs generally having higher abundance (e.g. *C. lanio*). This suggests that preferred resource quality of pigs is higher overall for those species, thereby appearing more attractive for colonisation. This is potentially due to microbial activity and the associated volatile organic compounds being released during decomposition. Microbes are the first species to colonise carrion and have a dynamic relationship with insects, since VOCs released by microbes are used by insects to detect carrion (Frederickx et al. 2012). The microbial community is inherently part of the carrion resource and a large aspect of the resource quality of carrion. As insects arrive on carrion, they also introduce additional microbes to the carrion, driving a shift in the VOCs released, thereby altering its attractiveness of the carrion (Tomberlin et al. 2011). Volatile organic compounds released during decomposition have been shown to be different between pigs and humans, suggesting that microbial communities may vary between these cadaver types, which would alter the resource quality (Knobel et al*.* 2019; Dawson et al. 2020). This difference is particularly true for donated human cadavers which are often sourced from hospitals and have been treated with numerous antemortem chemicals (Matuszewski et al. 2019; Dawson et al. 2020). More attractive VOCs may indicate higher nutritional output or digestibility of the carrion due to the microbial communities (Stavert et al. 2014; Kotzé et al. 2021).

The second distinct difference between pigs and humans was abundance peaking at different TBS values (e.g. *P. casei*). If abundance is similar, but the timings of phases of increase and decrease vary, then this may indicate that the resource quality changes at different rates, with the optimal resource quality state for each species, therefore varying between pigs and humans. The varying rates of resource quality are most likely due to the different decomposition rate exhibited by pigs and humans, with pigs often decomposing faster (Connor et al. 2017; Dautartas et al. 2018; Dawson et al. 2020). With quicker decomposition, pigs move through decay stages faster, giving associated species less time on pigs when resource quality is optimal.

## Conclusions and implications

Our study revealed how adult abundance patterns on carrion can provide insight into how species respond to carrion resource quality. Different species can arrive in similar abundances but at different times. This temporal partitioning likely leads to coexistence of species on carrion because species are adapted to exploiting carrion at different times or for different purposes. Contrastingly, different species can arrive at the same time but with differing magnitudes of abundances. In this case, coexistence is likely achieved because species are using the carrion resource for different purposes (feeding, oviposition, etc.). Abundance patterns also reveal the windows of optimal resource quality for carrion insect species. Species with short abundance peaks likely have narrow windows of opportunity when the resource quality is optimal and intraspecific competition is likely to be strong. In a forensic context, a species with a narrow window may be useful for PMI estimations. Species with long abundance peaks likely have a wide window of opportunity when resource quality is optimal. These species persist for longer on carrion, and therefore likely under lower levels of intraspecific competition. Our study highlights the many ways in which abundance data, as opposed to occurrence data, provide additional information relating to species ecological patterns. By analysing abundance patterns, we can examine species–resource interactions and the mechanisms that allow the coexistence of species on carrion. Our study only takes into consideration how insect respond directly to the carrion resource and does not examine other aspects of the necrobiome. To improve upon our findings, we suggest considering other factors such as ambient temperature, vertebrate scavengers and microbial communities. This approach might help to disentangle the relationship between carrion resource quality and insect community succession. To gain a more complete understanding of the complex interactions occurring on carrion, future research should examine how all aspects of the necrobiome respond to carrion resource quality.

## Supplementary Information

Below is the link to the electronic supplementary material.

Supplementary file1 (DOCX 332 KB)

Supplementary file2 (DOCX 26 KB)

Supplementary file3 (DOCX 18 KB)

## Data Availability

The datasets used and/or analysed during the current study are available from the corresponding author on reasonable request.

## Code availability

The code used during the current study are available from the corresponding author on reasonable request.
