# Supplementary material for: Insect abundance patterns on vertebrate remains reveal carrion resource quality variation
Source: Oecologia. 2022 Mar 16;198(4):1043–56. doi: 10.1007/s00442-022-05145-4 (PMC9056491; doi:10.1007/s00442-022-05145-4)
Supplement: Supplementary file 1 — Supplementary file1 (DOCX 332 KB) [file 442_2022_5145_MOESM1_ESM.docx]

**Supplementary material**


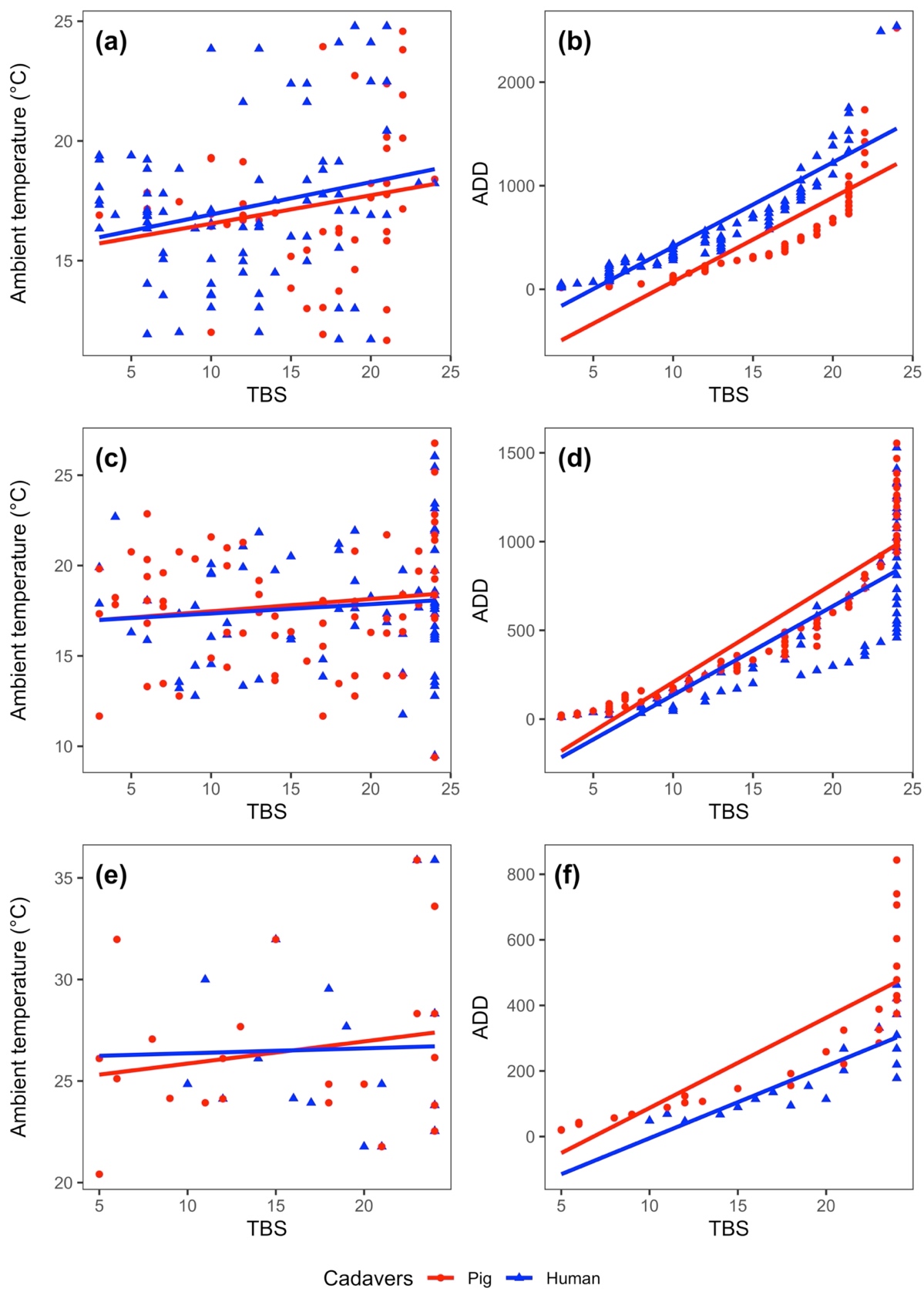


**Figure S1** Temperature plots comparing Winter A (a) ambient temperature and (b) accumulated degree days (ADD) against total body score (TBS), Winter B (c) ambient temperature and (d) ADD against TBS and Summer (e) ambient temperature and (f) ADD against TBS for pig (red) and human (blue) cadavers.
