# Supplementary material for: Insect abundance patterns on vertebrate remains reveal carrion resource quality variation
Source: Oecologia. 2022 Mar 16;198(4):1043–56. doi: 10.1007/s00442-022-05145-4 (PMC9056491; doi:10.1007/s00442-022-05145-4)
Supplement: Supplementary file 2 — Supplementary file2 (DOCX 26 KB) [file 442_2022_5145_MOESM2_ESM.docx]

**Supplementary material**

**Table S1** Mean ambient temperature (°C), humidity (%) and rainfall (mm) recorded for each TBS value across all cadaver types and seasonal experiments. Note, humidity data in Summer was not collected due an issue with the data logger.

| **Season** | **Cadaver** | **TBS** | **Mean ambient temperature (°C)** | **Mean humidity (%)** | **Mean rainfall (mm)** |
| --- | --- | --- | --- | --- | --- |
| Winter A | Pig | 3 | 16.9 | 40.78 | 0.6 |
|  |  | 6 | 17.5 | 41.23 | 0 |
|  |  | 8 | 17.46 | 47.75 | 0 |
|  |  | 10 | 16.79 | 67.27 | 0.52 |
|  |  | 11 | 16.51 | 64.18 | 0 |
|  |  | 12 | 17.52 | 46.3 | 0 |
|  |  | 13 | 16.69 | 44.07 | 0 |
|  |  | 14 | 16.99 | 57.29 | 0 |
|  |  | 15 | 14.52 | 63.9 | 0 |
|  |  | 16 | 14.22 | 70.66 | 0.1 |
|  |  | 17 | 16.28 | 73.84 | 0.1 |
|  |  | 18 | 15.41 | 49.25 | 0 |
|  |  | 19 | 17.74 | 32.7 | 0 |
|  |  | 20 | 17.93 | 34.72 | 0 |
|  |  | 21 | 17.21 | 48.39 | 0.09 |
|  |  | 22 | 21.52 | 48.49 | 0.04 |
|  |  | 24 | 18.4 | 39.99 | 1 |
|  | Human | 3 | 17.91 | 47.02 | 0.14 |
|  |  | 4 | 16.9 | 78.32 | 1.8 |
|  |  | 5 | 19.39 | 48.77 | 0 |
|  |  | 6 | 16.25 | 61.77 | 0.21 |
|  |  | 7 | 15.75 | 60.17 | 0.04 |
|  |  | 8 | 15.42 | 64.47 | 0 |
|  |  | 9 | 16.6 | 39.18 | 0 |
|  |  | 10 | 15.91 | 60.75 | 0.04 |
|  |  | 12 | 16.56 | 42.22 | 0.04 |
|  |  | 13 | 16.28 | 58.75 | 0.05 |
|  |  | 14 | 16 | 33.94 | 0 |
|  |  | 15 | 19.2 | 51.47 | 0.1 |
|  |  | 16 | 18.47 | 38.74 | 0.03 |
|  |  | 17 | 18.62 | 34.51 | 0 |
|  |  | 18 | 16.73 | 45.14 | 0.08 |
|  |  | 19 | 18.29 | 53.71 | 0 |
|  |  | 20 | 18.8 | 53.92 | 0.15 |
|  |  | 21 | 21 | 56.03 | 0.08 |
|  |  | 23 | 18.23 | 41.76 | 1 |
|  |  | 24 | 18.23 | 41.76 | 1 |
| Winter B | Pig | 3 | 18.9 | 57.53 | 0 |
|  |  | 4 | 22.69 | 57.7 | 0 |
|  |  | 5 | 16.3 | 55.23 | 0 |
|  |  | 6 | 16.97 | 55.46 | 0 |
|  |  | 8 | 14.7 | 81.95 | 0.2 |
|  |  | 9 | 15 | 64.24 | 0.13 |
|  |  | 10 | 17.95 | 60.59 | 0.8 |
|  |  | 11 | 16.49 | 66.49 | 0 |
|  |  | 12 | 18.1 | 63.5 | 0 |
|  |  | 13 | 17.76 | 81.65 | 0.5 |
|  |  | 14 | 19.73 | 70.91 | 0 |
|  |  | 15 | 17.5 | 82.68 | 0.07 |
|  |  | 17 | 14.33 | 45.86 | 0 |
|  |  | 18 | 19.88 | 40.99 | 0 |
|  |  | 19 | 18.83 | 44.13 | 0 |
|  |  | 20 | 18.27 | 40.39 | 0 |
|  |  | 21 | 17.1 | 41.05 | 0 |
|  |  | 22 | 16.02 | 59.88 | 0.04 |
|  |  | 23 | 18.12 | 66.94 | 0 |
|  |  | 24 | 18.36 | 52.11 | 0.98 |
|  | Human | 3 | 16.27 | 71.24 | 0 |
|  |  | 4 | 18.04 | 60.32 | 0 |
|  |  | 5 | 20.76 | 63.97 | 0 |
|  |  | 6 | 18.46 | 58.45 | 0 |
|  |  | 7 | 17.2 | 74.51 | 0.25 |
|  |  | 8 | 16.77 | 62.32 | 0 |
|  |  | 9 | 20.37 | 62.93 | 0 |
|  |  | 10 | 18.23 | 59.33 | 0 |
|  |  | 11 | 17.91 | 70.66 | 1 |
|  |  | 12 | 18.77 | 64.24 | 0 |
|  |  | 13 | 18.33 | 41.54 | 0 |
|  |  | 14 | 15.22 | 66.33 | 0.25 |
|  |  | 15 | 16.34 | 89.89 | 0 |
|  |  | 16 | 14.71 | 67.81 | 0.2 |
|  |  | 17 | 16.12 | 74.15 | 0.03 |
|  |  | 18 | 13.47 | 98.76 | 0.6 |
|  |  | 19 | 16.75 | 51.09 | 0.07 |
|  |  | 20 | 16.3 | 66.06 | 4 |
|  |  | 21 | 17.23 | 56.74 | 0 |
|  |  | 22 | 16.45 | 51.41 | 0 |
|  |  | 23 | 19.44 | 35.12 | 0 |
|  |  | 24 | 20.18 | 42.34 | 1.34 |
| Summer | Pig | 10 | 24.85 |  | 0 |
|  |  | 11 | 29.99 |  | 0 |
|  |  | 12 | 24.12 |  | 0 |
|  |  | 14 | 26.11 |  | 0 |
|  |  | 15 | 31.97 |  | 0 |
|  |  | 16 | 24.15 |  | 0 |
|  |  | 17 | 23.93 |  | 0 |
|  |  | 18 | 29.54 |  | 1.6 |
|  |  | 19 | 27.68 |  | 0 |
|  |  | 20 | 21.77 |  | 0.2 |
|  |  | 21 | 23.31 |  | 0.1 |
|  |  | 23 | 35.88 |  | 0 |
|  |  | 24 | 26.46 |  | 0 |
|  | Human | 5 | 23.26 |  | 0 |
|  |  | 6 | 28.54 |  | 0 |
|  |  | 8 | 27.06 |  | 0 |
|  |  | 9 | 24.15 |  | 0 |
|  |  | 11 | 23.93 |  | 0 |
|  |  | 12 | 25.12 |  | 0 |
|  |  | 13 | 27.68 |  | 0 |
|  |  | 15 | 31.97 |  | 0 |
|  |  | 18 | 24.39 |  | 0 |
|  |  | 20 | 24.85 |  | 0 |
|  |  | 21 | 21.77 |  | 0.2 |
|  |  | 23 | 33.36 |  | 0 |
|  |  | 24 | 26.73 |  | 0 |
