# Supplementary material for: Insect abundance patterns on vertebrate remains reveal carrion resource quality variation
Source: Oecologia. 2022 Mar 16;198(4):1043–56. doi: 10.1007/s00442-022-05145-4 (PMC9056491; doi:10.1007/s00442-022-05145-4)
Supplement: Supplementary file 3 — Supplementary file3 (DOCX 18 KB) [file 442_2022_5145_MOESM3_ESM.docx]

**Supplementary material**

**Table S2** GAM results for each species model. Significance donated by <0.001 (***), <0.01 (**), <0.05 (*) and non-significance (-). Total abundance for each species on human and pig cadavers also displayed.

|  | **Species** | **R-sq (adj)** | **Deviance (%)** | **Cadaver type** | **Human** | **Pig** | **Human**  **abundance** | **Pig**  **abundance** |
| --- | --- | --- | --- | --- | --- | --- | --- | --- |
| **Diptera** | *Piophila casei* | 0.0851 | 31.5 | - | *** | *** | 2505 | 1896 |
|  | *Chrysomya nigripes* | 0.0986 | 45.2 | *** | ** | * | 88 | 1880 |
|  | *Australophyra rostrata* | 0.0496 | 35.7 | *** | *** | *** | 344 | 1020 |
|  | Phoridae | 0.183 | 28.4 | *** | *** | *** | 337 | 954 |
|  | *Chrysomya varipes* | 0.116 | 27.4 | *** | *** | * | 304 | 754 |
|  | *Chrysomya rufifacies* | 0.0651 | 24.5 | *** | ** | - | 154 | 615 |
|  | *Chrysomya incisuralis* | 0.142 | 44.1 | *** | * | *** | 508 | 27 |
|  | *Dichaetomyia* sp. | 0.0463 | 20.1 | *** | - | * | 48 | 346 |
|  | *Calliphora* | 0.238 | 18.6 | ** | - | *** | 71 | 196 |
| **Coleoptera** | *Saprinus cyaneus cyaneus* | 0.163 | 52.9 | - | *** | *** | 2547 | 1437 |
|  | *Creophilus erythrocephalus* | 0.163 | 37 | - | *** | *** | 211 | 194 |
|  | *Necrobia rufipes* | 0.251 | 64.3 | - | *** | - | 375 | 28 |
|  | *Omorgus quadrinodosus* | 0.095 | 38.1 | - | *** | *** | 175 | 121 |
|  | *Creophilus lanio* | 0.256 | 39.3 | - | ** | *** | 188 | 50 |
| **Hymenoptera** | *Rhytidoponera metallica* | 0.0415 | 8.05 | *** | - | - | 72 | 158 |
|  | *Crematogaster* sp. | 0.164 | 38.3 | ** | *** | *** | 2711 | 867 |
|  | *Aphaenogaster longiceps* | 0.0357 | 6.6 | - | - | *** | 1218 | 636 |
|  | *Nasonia vitripennis* | 0.0845 | 26.5 | *** | ** | - | 836 | 630 |
